# Supplementary material for: Ipragliflozin Ameliorates Endoplasmic Reticulum Stress and Apoptosis through Preventing Ectopic Lipid Deposition in Renal Tubules
Source: Int J Mol Sci. 2019 Dec 26;21(1):190. doi: 10.3390/ijms21010190 (PMC6981520; doi:10.3390/ijms21010190)
Supplement: Supplementary file 1 [file ijms-21-00190-s001.pdf]

Supplementary Figure S1.

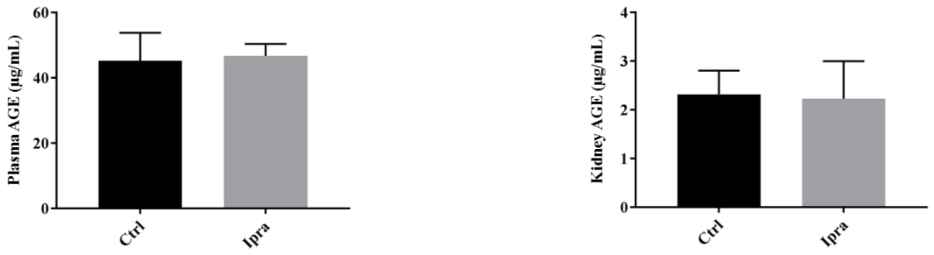

(**a**) (**b**)

Supplementary Figure S1. The levels of advanced glycation end product in plasma and kidney homogenates. The levels were measured by ELISA in the (a) plasma or (b) kidney tissues from FLS-*ob*/*ob* mouse. For the measurement in kidney AGE, homogenates containing 100µg of protein was used. No significant differences were found between each group. Bars indicate average ± SEM. Ctrl, control group; Ipra, Ipragliflozin group.
